# LB16. Surveillance Rates of Cefiderocol Heteroresistance Correlate With All-cause Mortality in the APEKS-NP and CREDIBLE-CR Trials

**DOI:** 10.1093/ofid/ofab466.1652

**Published:** 2021-12-04

**Authors:** Jacob E Choby, Tugba Ozturk, Sarah W Satola, Jesse T Jacob, David Weiss

**Affiliations:** 1 Emory University School of Medicine, Atlanta, GA; 2 Emory University, Atlanta, Georgia

## Abstract

**Background:**

Cefiderocol is a recently FDA approved, novel siderophore beta-lactam antibiotic. Conventional antimicrobial susceptibility testing (AST) suggests that a variety of Gram-negative pathogens, including carbapenem-resistant (CR) isolates, are overwhelmingly susceptible to cefiderocol.. This antibiotic performed well in the APEKS-NP trial for the treatment of nosocomial pneumonia caused largely by carbapenem susceptible isolates. However, in the CREDIBLE-CR trial involving exclusively CR Gram-negative bacteria, cefiderocol was associated with a higher rate of all-cause mortality. We hypothesized one explanation for these discrepant data might be undetected cefiderocol heteroresistance (HR).

HR is a form of antibiotic resistance in which an isolate harbors a minority resistant subpopulation of cells co-existing with a majority susceptible population, and is often undetected by standard AST. Isolates exhibiting undetected HR, with as low as 1 in 1 million resistant cells, can cause treatment failure in *in vivo* models.

**Methods:**

We quantified HR to cefiderocol by population analysis profile (PAP) of 161 *Acinetobacter*, 180 *Klebsiella*, and 108 *Pseudomonas* isolates collected in Georgia, USA.

**Results:**

We observed CR isolates exhibited a high frequency of HR, which was largely undetected by standard AST, and correlated with all-cause mortality in the CREDIBLE-CR study (Table). Carbapenem-susceptible isolates exhibited no or low rates of cefiderocol HR (Table). Cephalosporin-resistant bacteria mostly exhibited increased rates of cefiderocol HR, but below those of CR strains. These differences in rates of cefiderocol HR correlated with the mortality data from the APEKS-NP and CREDIBLE-CR trials, across the bacterial species tested (Table).

Table: Surveillance rates of cefiderocol heteroresistance correlate with all-cause mortality in the APEKS-NP and CREDIBLE-CR trials

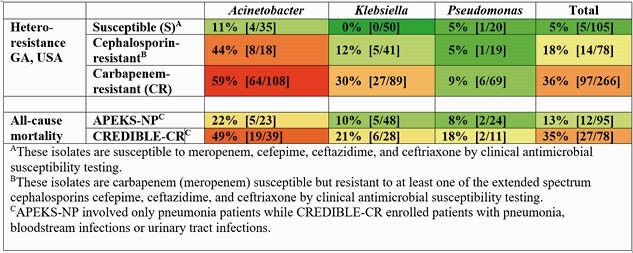

**Conclusion:**

These data suggest that the lower rates of cefiderocol HR in carbapenem-susceptible isolates that predominated the APEKS-NP trial may explain the enhanced efficacy of the drug in that study as compared to the CREDIBLE-CR trial. Importantly, the widespread, undetected cefiderocol HR observed among CR pathogens may explain the discordance between this drug’s excellent *in vitro* susceptibility profile and increased patient mortality in the CREDIBLE-CR trial.

**Disclosures:**

**All Authors**: No reported disclosures

